# First theoretical determination of relative biological effectiveness of very high energy electrons

**DOI:** 10.1038/s41598-021-90805-3

**Published:** 2021-05-27

**Authors:** Rachel Delorme, Thongchai A. M. Masilela, Camille Etoh, François Smekens, Yolanda Prezado

**Affiliations:** 1 Univ. Grenoble Alpes, CNRS, Grenoble INP, LPSC-IN2P3, 38000 Grenoble, France; 2 Imagerie et Modélisation en Neurobiologie et Cancérologie (IMNC), CNRS Univ Paris-Sud, Université Paris-Saclay, 91400 Orsay, France; 3 Institut Curie, Orsay Research Centre, CNRS UMR3347, INSERM U1021, University Paris Saclay, Orsay, France; 4 Dosisoft, R&D Medical Physics, Cachan, France

**Keywords:** Biological physics, Radiotherapy, Computational science

## Abstract

Very high energy electrons (VHEEs, E > 70 MeV) present promising clinical advantages over conventional beams due to their increased range, improved penumbra and relative insensitivity to tissue heterogeneities. They have recently garnered additional interest in their application to spatially fractionated radiotherapy or ultra-high dose rate (FLASH) therapy. However, the lack of radiobiological data limits their rapid development. This study aims to provide numerical biologically-relevant information by characterizing VHEE beams (100 and 300 MeV) against better-known beams (clinical energy electrons, photons, protons, carbon and neon ions). Their macro- and microdosimetric properties were compared, using the dose-averaged linear energy transfer ($\overline{{L_{d} }}$) as the macroscopic metric, and the dose-mean lineal energy $\overline{{y_{d} }}$ and the dose-weighted lineal energy distribution, *yd(y)*, as microscopic metrics. Finally, the modified microdosimetric kinetic model was used to calculate the respective cell survival curves and the theoretical RBE. From the macrodosimetric point of view, VHEEs presented a potential improved biological efficacy over clinical photon/electron beams due to their increased $\overline{{L_{d} }}$. The microdosimetric data, however, suggests no increased biological efficacy of VHEEs over clinical electron beams, resulting in RBE values of approximately 1, giving confidence to their clinical implementation. This study represents a first step to complement further radiobiological experiments.

## Introduction

Electron beams have been used to treat cancer and other diseases for over half a century^1^. Currently, most clinical electron beams have energies ranging from approximately 5 to 20 MeV. However, these beams are not suitable for treatment of deep-seated tumors, primarily due to their short range and substantial lateral scattering. Very high-energy electrons (VHEEs, E > 70 MeV) have been recently proposed^2^ as a novel approach for cancer radiation therapy (RT). Among the potential dosimetric advantages of VHEEs over photons, are the increased practical range and improved penumbra for the treatment of deep-seated tumors^2,3^. Furthermore, the absence of electronic disequilibrium at interfaces for VHEE therapy avoids significant dose variations arising at the boundaries of tissues with different densities, or at the interface between tissues and air cavities or bones^4,5^. Finally, recent treatment planning studies have demonstrated the systematic superiority of intensity-modulated VHEE therapy over state-of-the-art volumetric modulated arc therapy (VMAT) in decreasing the organ-at-risk doses for a number of anatomic tumor sites^6–8^. This superiority holds true for proton irradiations for the specific cases of head and neck cancers^8^. VHEE radiation therapy thus presents an intriguing option to treat cancer. The present-day technical limitations of the transfer of VHEEs to clinics resides in the ability to develop compact and possibly cost-effective facilities. These facilities should be capable of assessing complex irradiation schemes, ensuring reproducible dose delivery, and for which adapted dosimetry protocols would be needed. Recent advances in both compact high-gradient radio-frequency (RF)-based^9–11^ and laser-plasma wakefield^12–14^ accelerator technologies, in tandem with advanced magnetic beam focusing techniques to further improve the dose delivery^15–17^, gives confidence to the notion that clinically-compatible VHEE sources will be available in the near future. Nevertheless, such beams present dosimetry challenges due to the increased electron energy and very short pulse lengths (ns to fs) compared to current clinical electron accelerators (µs), thus inducing much higher dose-rates within the pulses. Passive dosimetry, using radiochromic films, has been proposed for VHEEs with promising results^18^, but solutions still need to be developed for real-time dosimetry^3,19,20^.

Recently, the use of ultra-high dose-rate irradiations have garnered increased interest as it has been demonstrated that they can induce the so-called “FLASH-effect”, namely a remarkable reduction of normal tissue complication probability compared to conventional dose rate regimes. The FLASH effect has been demonstrated in numerous animal studies^21,22^, and a first patient (skin melanoma) has already been treated^23^. Although some FLASH effects have been observed with photon^24^ and proton^25^ beams, the most established demonstrations were obtained using low-energy electron facilities (< 20 MeV), thus limiting their current potential application to superficial tumors. The use of VHEEs could address this limitation of penetration depth, thereby allowing the exploitation of the FLASH effect for the treatment of deep-seated tumors. VHEEs could thus be a very promising solution for FLASH applications, as long as the technology allows it. Developments such as the PHASER project, aimed at providing a clinical VHEE accelerator, makes progress in that direction^10^.

Moreover, because VHEE beams can be scanned electromagnetically down to very small diameters (< mm), it can also be of great interest for Spatially Fractionated Radiotherapy (SFR) approaches such as Grid^26^ or minibeam^27^ radiation therapy.

Since VHEEs are able to induce nuclear reactions, the secondary nuclear products generated could lead to an increase in the relative biological effectiveness (RBE). Previous theoretical studies have determined that secondary neutron production yield was relatively low^18^. To date, however, there is very little radiobiological data available which evaluates the efficacy of VHEE beams for treatments. This is mainly due to a lack of dedicated research platforms. Very recently, Small et al.^28^ performed the first plasmid DNA irradiations carried out with VHEEs at the CLEAR user facility at CERN, to determine their efficacy to produce DNA damage. VHEE RBE evaluated in terms of double strand break yield was found to be close to 1 for dry plasmids and 1.1–1.2 for wet plasmids. This is an important first step towards evaluating the impact of VHEEs at the molecular scale. Exploring the radiobiological response of living cells and tissues with such new beams would be the next necessary step before any clinical trials. In the meantime, Monte Carlo (MC) approaches can provide biologically-relevant information in order to further elucidate where exactly VHEE beams are situated amongst other better-known beams for therapy.

In the present work, we propose a numerical approach based on macro- and micro-dosimetric MC calculations and the use of a biophysical model to evaluate the theoretical RBE of VHEE beams. The objective is to compare the characteristic properties of 100 MeV and 300 MeV VHEEs with clinically available beams (photons, clinical electrons, protons and carbon ions), and with a neon ion beam which presents a renewed interest for minibeam therapy^29,30^. As in previous ion beam studies^31–34^, we used the unrestricted dose-averaged linear energy transfer (LET), $\overline{{L_{d} }}$, as our macroscopic metric, since it has shown the best correlation to the biological effects of proton beams^35^. The depth dose and depth $\overline{{L_{d} }}$ profiles obtained in a water phantom were used as figures of merit to compare several beam types. The investigation of microdosimetric properties relies on dose distributions *yd(y)* in lineal energy y, and dose-mean lineal energies $\overline{{y_{d} }}$ calculated at characteristics depths also in line with previous ion-beam microdosimetry studies^36–38^. Finally, an estimation of the cell survival curves and theoretical RBE of each beam as compared to the reference photon beam was calculated using the lineal energy spectra obtained from the MC simulations as inputs to the microdosimetric kinetic model (MKM)^38–41^.

## Materials and methods

### Monte Carlo code

All simulations were performed with GATE^42,43^ version 8.2, a MC simulation platform based on Geant4^44^ (version 10.5) and dedicated to imaging and radiation therapy applications. The same physics list *QGSP_BERT_HP_EMY* was employed for all simulations. It is a classically used physics list in GATE examples of radiation therapy, presenting a good compromise between computation time and precision in micrometer-scale simulations. This physics package in particular, uses the Bertini cascade model and high-precision neutron physics for hadronic processes, as well as the option 3 of the standard electromagnetic package of Geant4 as is widely recommended for medical applications^45^. Different sets of simulation parameters were used for the macrodosimetric and microdosimetric studies, as described below.

### Simulation details of the macroscopic dose and dose-averaged LET studies

To provide a more comprehensive physical description of particle beams, we have evaluated both the absorbed dose and the unrestricted dose-averaged LET, $\overline{{L_{d} }}$. The unrestricted LET, as described in ICRU report n° 85^46^, represents the energy loss per unit length travelled by charged primary particles and is derived from the electronic stopping power. This concept does not take into consideration the finite volume of interactions in a practical case, or the fact that high-LET particles will contribute to a higher dose for a given length travelled. The $\overline{{L_{d} }}$ is therefore a more biologically-relevant quantity as it better represents the complexity of a mixed radiation field, with an evolving energy spectrum in depth, in which each particle type and energy contribute to a particular dose in a finite target^32^. It is calculated as follows:

1 $$\begin{array}{*{20}c} {\overline{{L_{d} }} \left( z \right) = \frac{{\mathop \smallint \nolimits_{0}^{\infty } S_{el} \left( E \right)D\left( {E,z} \right)dE}}{{\mathop \smallint \nolimits_{0}^{\infty } D\left( {E,z} \right)dE}}} \\ \end{array}$$

where S_el_(E) is the electronic stopping power of primary charged particles with kinetic energy E and D(E,z) is the absorbed dose deposited by primary charged particles with kinetic energy E, at location z. It can be noted that this is a deterministic quantity.

The 3-dimensional dose and $\overline{{L_{d} }}$ distributions were collected using the Dose and LET actors of GATE respectively. Simulations were performed in a voxelized water phantom of 10 × 10 × 10 cm^3^, with a resolution of 100 µm in the beam direction, and 1 mm in both transverse directions. In practice, the calculation of $\overline{{L_{d} }}$ is performed in GATE as follows: at each simulation step for a particle of kinetic energy *E*_*k*_ depositing an energy *E*_*d*_ in a voxel, *E*_*d*_ is multiplied by the tabulated stopping power for the aforementioned particle in the material of the voxel (in this case water). This product is cumulated over all particles and all simulation steps in which energy is deposited in the voxel, and at the end of the simulation, this sum is divided by the sum of the *E*_*d*_ deposited by each particle in the same voxel.

The beam source was a square surface of 2 × 2 cm^2^ and particles were sent toward the water phantom with no angular divergence. The following mono-energetic beam types and energies were used: 1.25 MeV photons (i.e. mean energy of the 2 rays of ^60^Co); 5, 20, 100 and 300 MeV electrons; 105 MeV protons; 194.2 MeV/nucleon ^12^C ions and 262 MeV/nucleon ^20^Ne ions. The energies of the protons and heavy ions were chosen such that the Bragg peak was at the depth of a tumor centered in the brain (~ 8 cm).

The choice of simulation tracking parameters, such as the *production cut* and the *step limiter* values, may impact the $\overline{{L_{d} }}$ results^32^. The *production cut* determines the minimum penetration range in media above which secondary particles will be effectively transported in the simulation, while the *step* limit allows the avoidance of geometric border effects in small structures by forcing the particles having a mean-free path superior to this value to make an additional step. Based on preliminary simulation studies, we fixed the *production cut* and the *step limiter* values to the voxel size in the beam direction, i.e. 100 µm, which was the best compromise between high precision and reasonable computation time. This is in line with the recommendation made by Guan et al. for the step limit in $\overline{{L_{d} }}$ calculations^32^.

Between 10^9^ and 10^10^ primary particles were sent, according to the beam type, in order to obtain an uncertainty on dose deposition lower than 1% along the central axis profile in depth. The uncertainties on dose were calculated from the method of Chetty et al.^47^ for multicore MC-based simulations. For the $\overline{{L_{d} }}$ profiles, the values associated with doses lower than 0.1% of the max dose were not displayed due to large fluctuations on the calculated ratio. This concerns only the 5 MeV electron and 105 MeV proton beams due to their ranges being shorter than the chosen plotting depth of 10 cm, for which the uncertainty on dose corresponding to the abovementioned limit is about 5%. The statistical uncertainties on $\overline{{L_{d} }}$ values were approximated by doubling the uncertainties on doses in the same voxels, given that the dose appears twice in the equation of $\overline{{L_{d} }}$.

### Simulation details of the microdosimetric study

Since the discontinuous nature of energy transfers at the micrometric scale (typically the cellular scale) is not fully considered in the LET and macroscopic dose concepts, we used another formalism to consider the stochastic aspect of the irradiations. Microdosimetry is a recommended method for characterizing radiation quality when the biological effectiveness under test is not well known, as is the case for VHEE beams. In such situations, the radiation beams are described by their lineal energy probability distributions. Lineal energy *y* (expressed in keV/µm) is defined in ICRU report number 36^48^ as the energy imparted, *ε*, to matter in a given volume divided by the mean chord length, $\overline{l}$, of the volume:

2 $$\begin{array}{*{20}c} {y = \frac{\varepsilon }{{\overline{l}}}} \\ \end{array}$$

Since lineal energy *y* varies from one event to another, average values are often reported together with probability distribution functions in order to characterize the irradiation at a given point. In particular, the dose-mean lineal energy,$\overline{{y_{d} }}$, is a commonly used quantity, defined as follows^48^:

3 $$\begin{array}{*{20}c} {\overline{{y_{d} }} = \mathop \smallint \limits_{0}^{\infty } yd\left( y \right)dy = \frac{{\mathop \smallint \nolimits_{0}^{\infty } y^{2} f\left( y \right)dy}}{{\mathop \smallint \nolimits_{0}^{\infty } yf\left( y \right)dy}}} \\ \end{array}$$

where *f(y)* and *d(y)* are the probability and dose density distributions of lineal energy, respectively. $\overline{{y_{d} }}$ can be seen as the microdosimetric analogue of $\overline{{L_{d} }}$.

**Figure 1:**
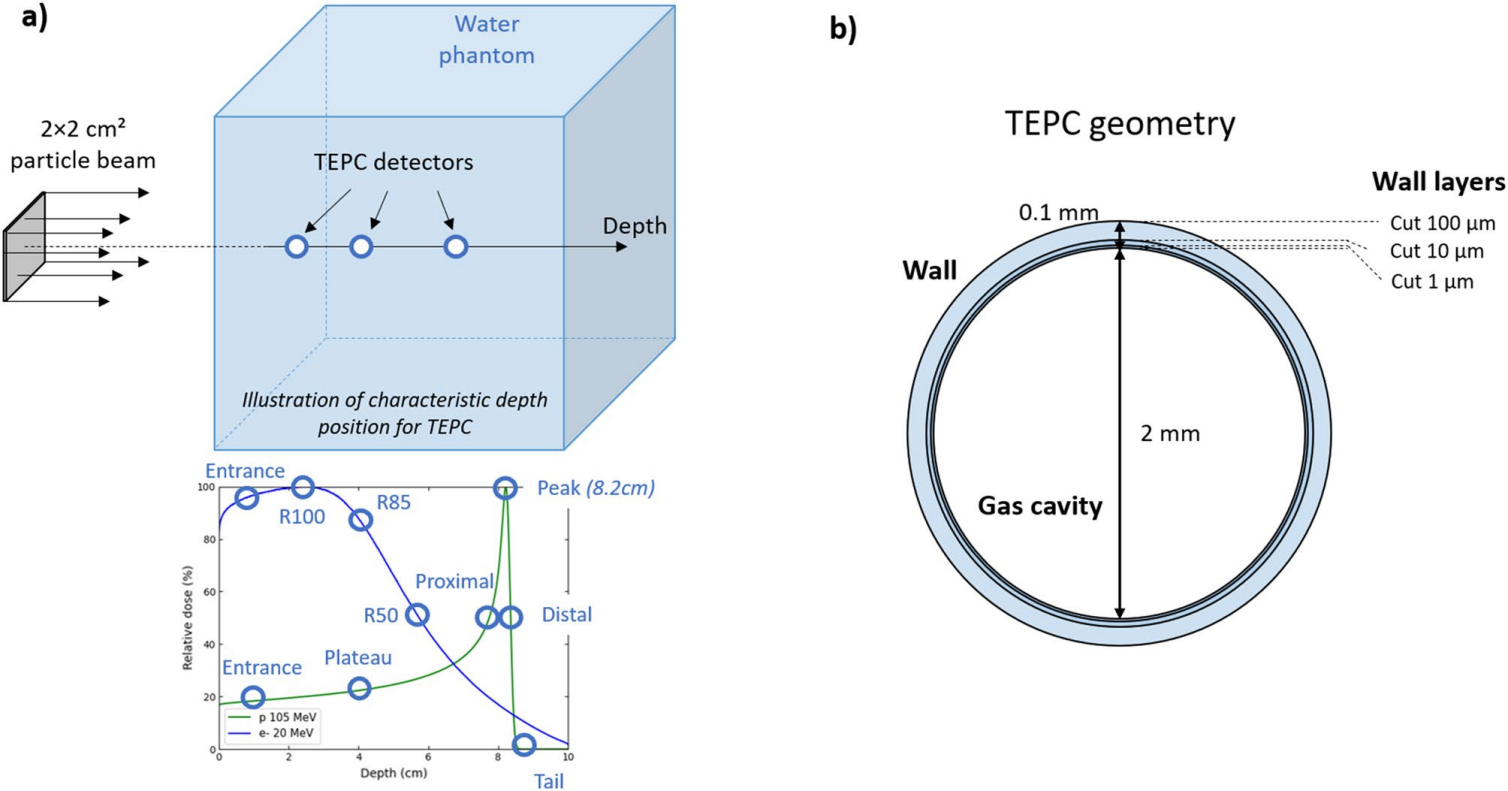
Illustration of (**a**) simulation geometry of particle beam sent in a water phantom and TEPC actors positioned at characteristics depth, and (**b**) geometry of the TEPC detector and cuts applied to each wall layer.

In our simulation study and in accordance with previous microdosimetric studies^31,32,34,36^, we chose to compare the dose-weighted lineal energy distributions, *yd(y)*, as well as the dose-mean lineal energy $\overline{{y_{d} }}$, so as to characterize all beam types at different characteristic depths. The f(y) distributions were collected thanks to the Tissue Equivalent Proportional Counter (TEPC) actor of GATE, and the other calculated microdosimetric quantities were thus derived from the aforementioned distributions according to Eq. (3). As illustrated in Fig. 1, the simulations consist of 2 × 2 cm^2^ beams of the same particle types as described in the macroscopic study, sent in a water phantom. TEPC detectors were positioned at characteristics depths along the central propagation axis in order to collect the required f(y) spectra. We designed the geometry of the TEPC actor according to the recommendations of the GATE collaboration, extensively discussed elsewhere^49^. The TEPC geometry is based on a model of a spherical TEPC gas detector (similar to the one used by Kase et al.^39^ in their experiment, the LET-1/2 TEPC detector of Far West Technology, Inc.), whose operation mode is similar to that of a classical ionization chamber but with a sensitive volume filled with a low-pressure tissue-equivalent gas, instead of air^50^. This particularity allows the detector to mimic the shape and composition of tiny biological structures, typically of 1 µm size. Figure 1B shows the GATE geometry corresponding to the TEPC detector as used in our simulations. The wall of the TEPC actor geometry was filled with water with total thickness of 0.1 mm, and consists of 3 concentric spherical layers of radius 1.1, 1.01 and 1.001 mm, respectively. The inner sensitive volume is a sphere of 2 mm diameter, filled with a mix of propane (C_3_H_8_), dioxide (O_2_) and nitrogen gas, with a pressure of 277 mbar which emulates interactions within a 1 µm diameter sphere of tissue-equivalent material. The production *cuts* and *step limiter* values in each volume were chosen as 1 mm in the water phantom, 100 µm, 10 µm and 1 µm in the first, second and third layers of the TEPC wall, respectively, and 1 µm in the sensitive TEPC volume. This multilayer configuration allowed the optimization of both the simulation time in the whole water phantom volume, and the precision of particle transportation for the determination of the lineal energy spectra. Smekens et al.^49^ demonstrated that this simulation configuration correctly reproduces the lineal energy spectra f(y) when compared against the measured data published by Kase et al.^39^ for a proton beam.

All lineal energy spectra *f(y)* obtained from the TEPC actors were normalized by event and collected according to a logarithmic scale of y values, with 150 bins ranging from 0.01 keV over 7 orders of magnitude. The characteristic depths for *f(y)* measurements were chosen differently according to the particle types, as illustrated in Fig. 1 – A). For proton and heavy-ion beams, TEPC actors were positioned at the entrance (1 cm depth), in the plateau region (4 cm depth), at the Bragg peak position (8.2 cm), in the proximal and distal dose fall-off regions (50% of peak dose), and in the fragmentation tail region. For photon and electron beams, TEPC actors were positioned at the entrance (1 cm depth or before max dose depth), 100%, 85% and 50% of maximal depth-dose corresponding to R100, R85 and R50, respectively. The water phantom is larger for the microdosimetric study compared to the LET study, with dimensions of 40 × 40 × 40 cm^3^, in order to reach the desired characteristic depths for VHEE beams. Since these characteristic depths may correspond to very different distances in water according to the beam type and energy, additional TEPCs were positioned at 4 cm and 8.2 cm in order to compare the microdosimetry spectra of all beam types at the same depth.

### Radiobiological calculations with MKM

The calculation of cell survival curves and theoretical RBE of each beam as compared to the photon ^60^Co reference was done using lineal energy spectra provided by the MC simulations and the MKM. The MKM is a biophysical model, developed in the 90’s by Hawkins et al.^41^, that allows the evaluation of RBE values for a given test radiation using biological data of a reference radiation, and physical data such as the *f(y)* spectra. Previous work by Kase et al.^39,40^ demonstrated that this model is capable of reproducing cell-survival curves of human salivary gland (HSG) tumor cell lines from microdosimetric spectra measured with a TEPC detector in various irradiation beams (photon, protons, and ion beams). The expression of cell survival fraction, S, based on the Linear Quadratic (LQ) model can be defined according to the MKM with the following relation^38^:

4 $$\begin{array}{*{20}c} {S = e^{{\left( { - \alpha_{MKM} D - \beta D^{2} } \right)}} = e^{{\left( { - \left( {\alpha_{0} + \frac{\beta }{{\rho \pi r_{d}^{2} }}\overline{y*} } \right)D - \beta D^{2} } \right)}} } \\ \end{array}$$

where *rd* is the mean radius of the sensitive domain studied (in µm), ρ is the density of tissue (i.e. 1 g/cm^3^ here), *D* is the total absorbed dose in gray (Gy), β is a biological parameter corresponding to the probability of inducing sublethal lesions in the LQ model (Gy^-2^), and α_0_ is a biological parameter calculated from a combination of reference irradiations and high-LET irradiations for a given cell-line. These parameters are independent of the tested irradiation beam and can be determined for a specific cell line. The parameters α0, *y*0, β and *rd* used in this study were issued from Kase et al.^39^, corresponding to biological data of the HSG tumor cell line. Finally, $\overline{y*}$ is the lineal energy weighted in dose and corrected by the saturation parameter *y*_*0*_, expressed as:

5 $$\begin{array}{*{20}c} {\overline{y*} \left( {keV \cdot \mu m^{ - 1} } \right) = \frac{{y_{0}^{{2\smallint 1 - e^{{ - \left( {\frac{y}{{y_{0} }}} \right)^{2} }} f\left( y \right)dy}} }}{\smallint yf\left( y \right)dy}} \\ \end{array}$$

We can see that $\overline{y*}$ is the lone quantity of the survival fraction expression that depends on the physical characteristics of the beams. Hence this model allows the estimation of a cell survival fraction, and an RBE calculation for different tested beam qualities, solely from the calculated values of lineal energy y and lineal energy frequency distribution f(y) from the MC simulations. We have thus calculated theoretical cell survival curves of HSG cell lines for all beam qualities and all depths in water (corresponding to TEPC positions) previously described. We also calculated theoretical RBE_10_ values as the ratio of the dose needed with the calculated gamma rays of ^60^Co to obtain 10% of cell survival to the dose of the tested beam for the same survival fraction.

The statistical uncertainties on calculated microdosimetric quantities ($\overline{{y_{d} }}$, $\alpha_{MKM}$, survival fractions and RBE_10_) were estimated from the standard error *σ*_*i*_ associated to each bin content *y*_*i*_ of the initial calculated spectra *f(y)*. For each configuration, a thousand f(y) spectra were generated from a normal distribution of mean value *y*_*i*_ and standard deviation *σ*_*i*_ for each bin. The final uncertainty on the microdosimetric quantity, σ, is the standard deviation of the 1000 values calculated for the 1000 randomly generated f(y) spectra.

## Results

### Macrodosimetry study: dose and $\overline{{L_{d} }}$ profiles

**Figure 2:**
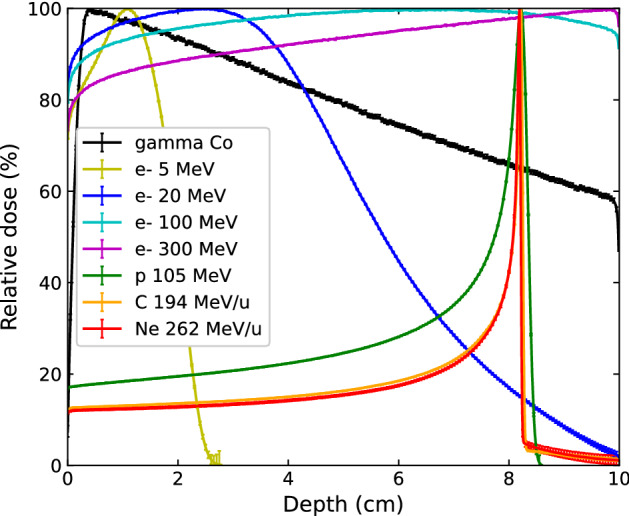
Depth-dose profiles in water obtained for different photon, clinical electrons, VHEE and ion beams of size 2 × 2 cm^2^. Doses lower than 0.1% of max dose are not shown. Uncertainties on absorbed doses in central axis voxels are less than 1%.

**Figure 3:**
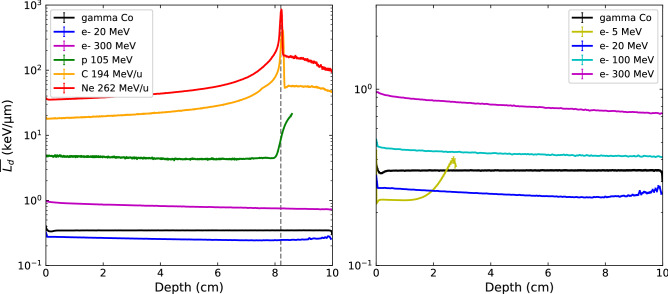
Dose-averaged LET profiles in depth to water for photon, clinical electrons, VHEE and ion beams (left), and details of the $\overline{{L_{d} }} { }$ results for all electron beam energies (right). The vertical grey dashed line marks the Bragg peak position. $\overline{{L_{d} }} { }$ values associated to doses lower than 0.1% of max dose are not shown to keep statistical relevance. Uncertainties on $\overline{{L_{d} }} { }$ values in central axis voxels are less than 2%.

Results of depth-Dose and depth-$\overline{{L_{d} }}$ profiles, obtained with the simulation parameters detailed in section “Simulation details of the macroscopic dose and dose-averaged LET studies”, can be found in Figs. 2 and 3 respectively. Doses and $\overline{{L_{d} }} { }$ values associated with doses lower than 0.1% of the max dose are not shown in order to maintain the plots' statistical relevance, as they correspond to the end of range values for the 5 MeV electron and proton beams. Uncertainties were calculated as described in the above-mentioned section, and were found to be a maximum of 1% and 2% for absorbed doses and $\overline{{L_{d} }} { }$, respectively, in the central axis voxels of the plotted points. Although the present study concerns only broad beams, we have limited calculations to 10 cm depth in water to focus on irradiation parameters relevant for brain tumors, in coherence with previous studies of our team related to SFR treatment strategies^27,30^. As expected, VHEE beams present a flatter depth-dose profile than clinical electron beams and standard photon beams, with maximum values reached around 7 cm and beyond 10 cm in depth for 100 MeV and 300 MeV respectively. This dosimetry characteristic is often put forward as an argument in favor of VHEE beams in comparison to photon beams to treat deep-seated tumors, especially when using intensity modulation techniques^6,8^. Both carbon and neon ions have very similar relative depth-dose profiles with a very sharp pristine Bragg peak in comparison to protons.

In terms of $\overline{{L_{d} }} { }$ profiles, carbon-ion and neon-ion beams present very high LET values (> 200 keV/µm) and a similar behavior in depth, starting with a continuous increase of LET values up to a maximum around the Bragg-peak position (the maximum is shifted by a millimeter after the Bragg peak position for carbon-ions), a brutal decrease after the Bragg peak, and still higher LET values in the fragmentation tail in comparison to the plateau region. However, the neon-ion beam presents $\overline{{L_{d} }} { }$ values 3 times higher than carbon-ion beams. This result is in accordance with the work of Gonzalez et al.^30^ performed on minibeam irradiations and suggest that Ne-ion beams should induce higher biological effects than carbon-ion beams, for very similar physical dosimetric behaviour. In comparison, proton’s $\overline{{L_{d} }} { }$ values stay lower than 100 keV/µm up to the Bragg peak and are higher in the distal region. One can note that for proton, and to a lesser extent carbon-ions, the maximum LET value is shifted to a higher depth compared to the maximum of absorbed dose, while neon-ion doesn’t present this shift in depth, which could be an advantage for treatment planning optimization considering both quantities.

Photon and electron beams led to $\overline{{L_{d} }} { }$ values lower than 1 keV/µm, with almost no variation in depth, except for the 5 MeV electrons due to their very short range compared to the total depth studied. However, VHEE beams, and in particular 300 MeV electrons, appear to have much higher LET than clinical beams. The $\overline{{L_{d} }}$ values at 4 and 8.2 cm in depth are reported in Table 1. The corresponding ratio of $\overline{{ L_{d} }} { }$ values between 300 MeV electrons to other particles is 0.04, 0.2, 1.9, 3.2 and 2.4, at 4 cm depth in water, and 0.003, 0.09, 1.8, 3.1 and 2.2, at the Bragg peak position, for carbon ions, protons, 100 MeV electrons, 20 MeV electrons and photons respectively. This could suggest a potential higher biological efficacy of 300 MeV VHEE beams for cancer treatment than that of clinical photon or electron beams.

**Table 1:** Calculated values of $\overline{{L_{d} }}$ and $\overline{{y_{d} }}$ for all simulated particles of sufficient range, and of α_MKM_ and RBE_10_ for 20 MeV and 300 MeV electron, proton, carbon and neon ion beams at 4 and 8.2 cm in depth.

Particle	$\overline{{L_{d} }}$—4 cm	$\overline{{L_{d} }}$—8.2 cm	$\overline{{y_{d} }}$—4 cm	$\overline{{y_{d} }}$—8.2 cm
^60^Co gammas	0.345 ± *0.003*	0.346 ± *0.003*	1.85 ± *0.05*	1.85 ± *0.05*
20 MeV electrons	0.255 ± *0.001*	0.244 ± *0.001*	1.307 ± *0.006*	1.43 ± *0.01*
100 MeV electrons	0.431 ± *0.001*	0.420 ± *0.001*	1.265 ± *0.007*	1.273 ± *0.005*
300 MeV electrons	0.817 ± *0.001*	0.757 ± *0.001*	1.251 ± *0.006*	1.29 ± *0.02*
105 MeV protons	4.384 ± *0.009*	8.695 ± *0.02*	5.3 ± *0.6*	12.86 ± *0.08*
194 MeV/n Carbon ions	22.26 ± *0.03*	231.9 ± *0.4*	25.2 ± *0.2*	325.4 ± *0.4*
262 MeV/n Neon ions	43.8 ± *0.2*	762 ± *5*	45.73 ± *0.08*	990.7 ± *0.7*

Particle	α_MKM_—4 cm	α_MKM_—8.2 cm	RBE_10_—4 cm	RBE_10_—8.2 cm
20 MeV electrons	0.149 ± *0.001*	0.151 ± *0.001*	0.989 ± *0.001*	0.991 ± *0.001*
300 MeV electrons	0.148 ± *0.001*	0.148 ± *0.001*	0.988 ± *0.001*	0.988 ± *0.001*
105 MeV protons	0.173 ± *0.001*	0.307 ± *0.001*	1.023 ± *0.001*	1.233 ± *0.001*
194 MeV/n Carbon ions	0.430 ± *0.001*	1.160 ± *0.001*	1.446 ± *0.001*	2.934 ± *0.003*
262 MeV/n Neon ions	0.740 ± *0.001*	1.311 ± *0.001*	2.045 ± *0.002*	3.267 ± *0.004*

### Microdosimetry study

**Figure 4:**
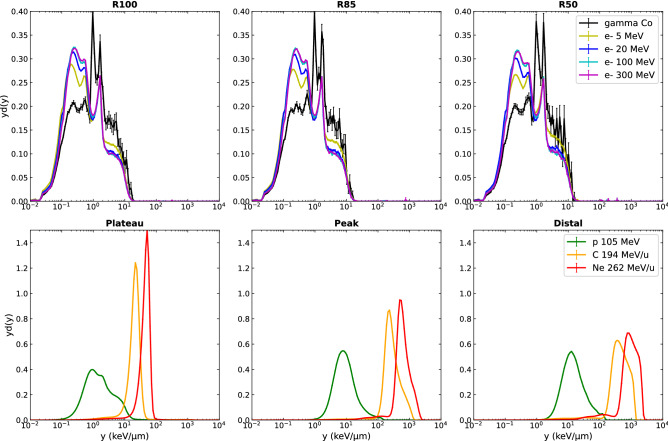
Comparison of microdosimetric spectra yd(y) for photon and electron beams (upper panel), and proton and ion beams (lower panel) calculated at characteristic depths.

As described in section “Simulation details of the microdosimetric study”, the lineal energy spectra *f(y)* and *yd(y)* were calculated using the TEPC actor of GATE, and used as figures of merit to represent the stochastic aspect of the irradiations at the microscopic scale, which is not fully considered in the LET concept. Indeed, *yd(y)* is an interesting quantity to quantity VHEEs (poorly known in terms of biological effects) against better-known radiation types, as it is expected that two radiation types having identical dose microdosimetric distributions should have the same RBE^36^*.* Figure 4 shows the resulting lineal energy *yd(y)* spectra obtained at different characteristic depth for the electron and photon beams on one side (upper row), and those obtained for ion beams on the other side (lower row). Error bars correspond to the standard error *σ*_*i*_ associated to each bin content *y*_*i*_ of the calculated spectra *f(y)*, propagated through the calculation of the *yd(y)* spectra (see section "Radiobiological calculations with MKM"). The choice of characteristic depths for ion beams (plateau, peak, distal) is made according to their relevance in observing the largest evolution of microscopic characteristics along the beam path, in coherence with other microdosimetric studies^36,37^. The characteristic depths for electron and photon beams (R100, R85, R50) were chosen to compare the different beams over their entire range because of their relevance in clinical dosimetry, although it corresponds to very different absolute depths in water according to the electron energy used.

**Figure 5:**
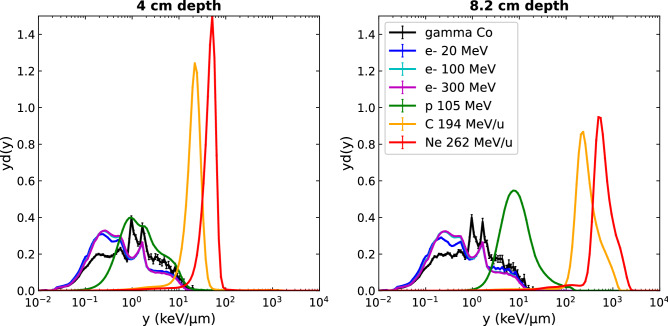
Comparison of microdosimetric lineal energy spectra yd(y) for all beam types at the same depths of 4 cm (plateau) and 8.2 cm (Bragg peak position) in water.

In order to compare all beam types at the same depth, Fig. 5 shows the dose-weighted *yd(y)* lineal energy distributions calculated at 4 cm and 8.2 cm depth in water.

The photon and electron beams, independent of their initial energy, present very similar calculated dose probability density *yd(y) spectra,* distributed broadly over 3 orders of magnitude of lineal energy values from 0.01 to 10 keV/µm, with no significant changes of spectra shapes with depth. Although in the same range, photon beam spectra have a different shape to the electron beams, showing slightly higher probabilities of high dose-weighted events, characterized by a higher amplitude in the right part of the lineal-energy spectra. Indeed, more energy is transmitted on average per photon interaction than per electron interaction, the latter resulting in more frequent events depositing less energy. According to these lineal energy distributions, no specific behavior of VHEE beams can be extracted in comparison to clinical electron energies. The *yd(y)* distributions for ion beams are characterized by a shift to higher lineal energy values for increasing depth, reaching maximum y values of about 100, 1000 and 2000 keV/µm for proton, carbon, and neon ion beams respectively. This shift toward higher lineal energies with depth is accompanied by an amplitude decrease and a broadening of the spectra, because of the lower dose weighting and, at the distal position, the additional contribution of the secondary particles coming from the fragmentation of carbon and neon ions. The higher the mass of the particle, the narrower the dose-weighted lineal energy spectra and the larger the shift to high lineal energy values. A shift of the spectra in high *y* values would represent an increase in the aggressiveness of the event, i.e. inducing more frequently lethal events. This clearly highlights and separates the microscopic behavior of typical low-LET particles (electron, photons and protons in the plateau region) with that of high-LET particles (heavier ions and proton in the Bragg peak region). In particular, the Ne-ions present very narrow spectra, extending over one order of magnitude of lineal energy values from 10 to 100 keV/µm in the plateau region, and from 200 to 2000 keV/µm in the Bragg peak region. The protons have intermediate behavior, with *yd(y)* spectra extending over two order of magnitude of lineal energy values, from 0.1 to 20 keV/µm in the plateau region, and from 1 to 100 keV/µm in the Bragg peak region. These simulated spectra are in good accordance with the measured *yd(y)* spectra obtained with a TEPC detector and published by Kase et al.^39^ for plateau and Bragg peak regions (although the initial proton beam energy was different in their experiment, 155 MeV, inducing slight changes in spectra amplitude). This validates the reliability of the GATE actor and physics models used to reproduce experimental microdosimetric beam characteristics.

**Figure 6:**
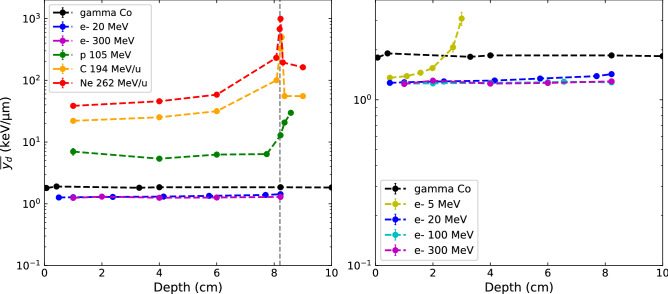
Dose-mean lineal energy profiles $\overline{{y_{d} }}$ in depth to water for photon, clinical electrons, VHEE and ion beams (left), and details of the $\overline{{y_{d} }}$ values for all electron energies (right). The vertical grey dashed line marks the Bragg peak position.

In order to complete the comparison of microdosimetric quantities, the dose-mean lineal energy $\overline{{y_{d} }}$ was calculated according to Eq. (3) for different depths in water and for each beam type, in order to act as the microdosimetric analogue to $\overline{{L_{d} }}$. Figure 6 shows the calculated $\overline{{y_{d} }}$ as a function of depth for all particle types (left), with a detailed plot of all electron beam energies and ^60^Co gammas (right). The statistical uncertainties were calculated as described in section "Radiobiological calculations with MKM", and were found to be less than 1% most of the time, except for photons (approximately 2.5%), 5 MeV electrons at the end of their range (up to 9%) and protons in the plateau region (up to 10%).

The values of $\overline{{L_{d} }}$ and $\overline{{y_{d} }}$, calculated at the depths of 4 and 8.2 cm are reported in Table 1. When comparing macroscopic $\overline{{L_{d} }}$ to these microscopic quantities $\overline{{y_{d} }}$, a first observation is that their evolution follows a very similar tendency in depth for all particle types. The absolute values of $\overline{{y_{d} }}$ in comparison to $\overline{{L_{d} }}$ are of the same order of magnitude for ion beams, however much higher $\overline{{y_{d} }}$ values are obtained for the photon and electron beams compared to $\overline{{L_{d} }}$ values. More importantly, VHEE beams do not display higher values when compared to clinical electron beams, and in fact have even lower values than photon beams. This marks a significant difference with the macroscopic approach, as $\overline{{L_{d} }}$ values were significantly higher for 300 MeV electrons in comparison to clinical photon or electron beams (see Table 1). For comparison, $\overline{{y_{d} }}$ values obtained at a depth of 4 cm are 5.4, 5.1, 1.5 and 1.2 times higher than $\overline{{L_{d} }}$ values for photons, 20 MeV electrons, 300 MeV electrons and protons respectively. Despite these absolute quantities not being directly comparable since the $\overline{{L_{d} }}$ was calculated in voxels with a minimum size of 100 µm while $\overline{{y_{d} }}$ was calculated in spherical volumes of 1 µm equivalent tissue, the relative comparison between particle types still highlights the important difference regarding the situation of VHEEs using the two dosimetric approaches. Hence, no increased biological effectiveness seems to be expected with VHEE beams in comparison to clinical electron beams from the microdosimetric point of view.

### Cell survival calculations and RBE

**Figure 7:**
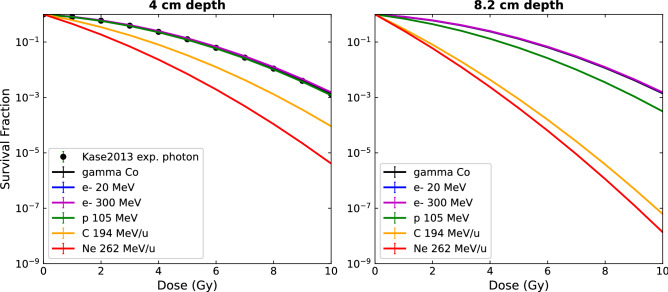
Theoretical survival curves calculated for different particles at a depth of 4 (left) and 8.2 (right) in colored lines. Experimental cell survival curve for HSG cells exposed to photons represented with black points^39^.

As described in section “Radiobiological calculations with MKM”, the cell survival curves were calculated using the simulated lineal energy spectra and the MKM model according to Eqs. (4) and (5). The biological model parameters α0, *y*0, β and *rd* were provided by Kase et al.^39^, with values of 0.13 Gy^−1^, 150 keV/μm, 0.05 Gy^−2^ and 0.42 µm, respectively, and corresponding to the biological data of the HSG tumor cell lines. Calculated survival curves at 4 and 8.2 cm depth are shown in Fig. 7, comparing photons, 20 MeV and 300 MeV electrons, protons, and heavier ions—all represented by colored lines. The error bars represent the statistical uncertainties on theoretical survival fractions, calculated as described in section "Radiobiological calculations with MKM", and were found to be less than 0.1%. The black points correspond to fitting parameters of the experimental HSG cells irradiated with 200 kV X-rays, extracted from Kase et al.^39^, using an α_MKM_ = 0.164 ± 0.008 Gy^−1^ and a β = 0.05 Gy^−2^.

The corresponding calculated $\alpha_{MKM}$ and RBE values for each beam type and depth are reported in Table 1. The RBE values were calculated using the calculated cell survival curve for ^60^Co gammas as the reference, in order to maintain consistency between the data obtained from the calculations. However, similar results would have been obtained by instead using the experimental cell survival curve for 200 kV X-rays, as the fitting parameters of both curves are very close ($\alpha_{MKM, Kase200kV}$ = 0.164 vs $\alpha_{MKM, Co}$ = 0.157). The statistical uncertainties on microdosimetric quantities were calculated from the standard errors on the bins of the f(y) histograms and then propagated through the subsequent equations. Uncertainties on $\overline{{L_{d} }}$ were calculated as the double of the one sigma dose uncertainties in the corresponding voxels.

In line with the microdosimetry study, cell survival curves of photon and electrons of any energy present no discernible differences, leading to an RBE of 1 for electrons beams, thus indicating a similar biological effect for all electron beams. In the left plot of Fig. 7, good accordance was found between both calculated and experimental survival curves for photon irradiations, illustrating the relevance of the calculations. Both Fig. 7 and the calculated RBE_10_ values illustrate the benefit that high LET particles have over traditional photon beams in terms of their enhanced cell killing capabilities for the same dose delivered. Interestingly, survival curves of carbon in comparison to neon ions are more separated in the plateau region than in the peak. This phenomena could be explained by an increased overkilling effect for Neon ions in the Bragg peak region, as their LET are much higher than 200 keV/µm, a limit above which an overkill effect has been reported for heavy ions^51^. The calculated RBE values associated with a 10% survival fraction were, as expected, increasing with the LET of the particles in depth, as well as increasing for higher Z. This is in agreement with the RBE values found in literature^52^. For example, we found an RBE of approximately 1.2 in the Bragg peak region for protons, which had also been reported in Paganetti et al.^53^. Furthermore, RBE values of about 3 for carbon and neon ions of 200 keV/µm have been reported for HSG cell line irradiations by Furusawa et al.^54^, in accordance with our calculations in the Bragg peak region.

## Discussion and conclusion

VHEE beams were proposed 20 years ago as an interesting alternative to photon beams for radiotherapy treatments, due to their improved penetration range, reduced lateral penumbra, and relative insensitivity to heterogenous tissues thus leading to decreased doses brought to organs at risks^2,5,6,8^. In addition, the possibility of scanning beams with magnetic collimation down to very small beam sizes, reaching potentially very high intensities, opens up their use in combination with new dose delivery approaches such as spatially fractionated radiotherapy^26,27^ or ultra-high dose rate therapy, inducing the so-called “FLASH” effect^21–23,55^. Both approaches have been shown to provide an increased tolerance of healthy tissues to high doses, and VHEE beams are of particular interest to provide such effects in deep tissues. However, due to the lack of dedicated radiobiological platforms currently available, the biological effect of such beams in living matter is still poorly known. MC approaches can provide biologically-relevant information on the situation of VHEE beams amongst better-known beams for therapy.

The present work provides a theoretical comparison of VHEE beams against clinically used photon, electron and ion beams, in terms of biologically relevant observables calculated following two separate macrodosimetric and microdosimetric approaches using the GATE MC platform. To our knowledge, it is the first time that such a study is performed for VHEE beams, up to the determination of a theoretical RBE. One key result of this study is that depending on the scale chosen (macro vs. micro) to evaluate the dosimetric quantities, different conclusions can be reached with regards to the biological efficacy of these high-energy electron beams compared to other beams.

From a macrodosimetric point of view, VHEE beams present a potential improved biological efficacy over clinical photon and electron beams, due to their increased $\overline{{L_{d} }}$ as shown in Fig. 3, a metric that correlate well to the biological effects observed for ion beams according to McMahon et al.^35^. Quantitatively, the ratio of $\overline{{L_{d} }}$ values for 300 MeV electrons to other beams was found to be 0.2, 1.9 and 3.2 for protons, 100 MeV electrons and 20 MeV electrons respectively. This result places VHEEs between electrons at clinical energies, and proton beams in the plateau region in terms of biologically-relevant macroscopic quantities.

In contrast, the microdosimetric data suggests no increased biological effectiveness of VHEE beams over clinical electron beams as no significant differences were found between their lineal energy spectra nor their $\overline{{y_{d} }}$ depth profiles, as shown in Figs. 4, 5 and 6. This conclusion was further illustrated by the fact that the cell survival curves for the VHEE beams were indistinguishable to those of the clinical beams, thus resulting in a theoretical RBE of ~ 1 for all electron energies. This result is in accordance with the first experimental evaluation of VHEE RBE published recently by Small et al.^28^ which was found to be close to 1, although plasmid DNA damage was used as the biological endpoint for the RBE calculation instead of cell survival. Both results give confidence to the clinical implementation of VHEE radiotherapy, as the biological damage caused by VHEEs are expected to be similar to those caused by conventional radiotherapy modalities.

It is worth noting that the absolute values of both $\overline{{L_{d} }}$ and $\overline{{y_{d} }}$ may change with the size of the given volume. For example, it has been shown by Liamsuvan et al.^36^ that the $\overline{{y_{d} }}$ decreases if the target size increases from 10 to 100 nm for low-LET ions. We also have confirmed this tendency for low-LET particles by calculating the $\overline{{y_{d} }}$ obtained on target sizes of 1 µm and 10 µm with proton, photon and electron beams, and up to 100 µm with photon beams. The largest impact was for photon beams, with $\overline{{y_{d} }}$ values obtained that were 3.7 times larger for a target of 1 µm compared to one of 100 µm. In addition, Cortes-Giraldo et al.^31^ and Guan et al.^32^ have both warned about the influence of simulation parameters or target size on the $\overline{{L_{d} }}$ values calculated. This highlights the interest in providing complementary approaches, needed to characterize, at different levels, the new beams of interest, as was done in the present study for evaluation of VHEE beams.

It should be noted that this study was performed using a particular type of air-based TEPC detector implemented in GATE. Different types of TEPC detectors exist (see e.g. Table 1 in ref.^56^), with different materials (gas- or solid-based detectors) and different sensitive volume sizes (from 0.5 µm to 30 µm). One can expect significant differences in the microdosimetric quantities obtained according to the type of detector chosen to collect the *f(y)* spectra, with the aforementioned sensitive volume size being particularly influential. A comprehensive study was performed by Parisi et al.^56^, in which they demonstrated that despite the large variation in sizes and materials, good agreements were found between calculated $\overline{{y_{d} }}$ and RBE trends for the different detector types studied, with an average deviation of 0.8% and 5.7% for proton and ^12^C ion beams, respectively. We assume, therefore, that the choice of the TEPC detector would not significantly alter the conclusions of the present study regarding the biological effects expected for VHEEs, nor the relative comparisons of the microdosimetric quantities.

As there is currently no standard procedure for experimental or numerical microdosimetry studies, the present theoretical study aims to provide potential indicators, but only future biological experiments will allow concrete conclusions to be drawn on these new beams. In particular, the RBE values calculated in the present study are not intended to represent any FLASH-related biological effects, but rather the relative biological behavior that may arise in water based on the different physical parameters of each beam. FLASH leads to different effects in normal and tumoral tissues, implying other biological and chemical mechanisms which goes far beyond the claims of this simulation study. Additionally, macro- and microdosimetric quantities were calculated in water, without considering tissue heterogeneities that may appear in reality. Differences in tissue densities would impact the dosimetric properties of these beams, and potentially the microdosimetric quantities on which our theoretical radiobiological calculations are based. Nevertheless, one of the most important dosimetric advantage of VHEEs is their relative insensitivity to tissue heterogeneities as compared to photon and ion beams, making them more robust to potential calculation errors as experimentally demonstrated by Lagzda et al.^57,58^. Hence, the differences between simulated and realistic materials should not significantly impact our conclusions about VHEEs and their relative comparison with other beams.

Other challenges in accelerator technologies and dosimetry developments are to be overcome. RF-based accelerators can provide relatively compact and quasi monoenergetic VHEE beams up to 250 MeV, such as the CLEAR user research platform at CERN^11^. Several dosimetry experiments have already been performed on it which demonstrated the behavior of beam stability^3,11,58^. Interesting propositions were made to reduce the doses delivered to entrance tissue, an inherent problem of VHEEs, by focusing the beam at a certain depth in tissue, a configuration whose feasibility was demonstrated on CLEAR^16,17^. FLASH-compatible dose-rates should also be achievable on such VHEE RF-based accelerators^17,59^. New developments based on very high-gradient RF accelerator technologies (up to 100 MV/m) should make even more compact facilities available^9^, as proposed by the PHASER project^10^. Laser-plasma technologies may also result in a significant reduction in machine size and cost to produce VHEE beams in comparison to RF-based accelerators. Recent experimental studies have shown that such technologies are capable of delivering multi-incidences and complex intensity modulation irradiation schemes on millimeter-sized beam to treat deep seated tumors^13,14^. In terms of dosimetry, shot-to-shot charge and spectral fluctuations, in addition to the extremely short femtosecond pulses inherent to laser-plasma sources, may yield further difficulties in finding real-time dosimetry solutions, although several studies have shown that averaging over hundreds of pulses should be sufficient to ensure good precision and reproducibility when delivering doses to samples^13,14,18,60,61^. VHEE beam dosimetry will also have to cope with potential problems of small field dosimetry, particularly for magnetically-focused beams, and to a lesser extent, the increased energy of these beams when compared to currently used ionization chambers, which are calibrated for electrons up to 50 MeV^18,19^. Providing reference dosimetry protocols for VHEE beams and ultra-short high dose-rate pulses is a big challenge, which, for example, several European reference metrology institutes within the UHDpulse project are trying to answer^20^. All these new developments are promising advancements which may make VHEE beams clinically viable within the current decade.

In addition to VHEEs, neon ion beams, although not used clinically, were added to this study because of their renewed interest for minibeam therapy^29,30^. This provides complementary biologically-relevant data to the previous calculations by comparing, in particular, microdosimetric spectra and theoretical RBE in the plateau and the peak region of the neon beams, to classical beams of proton and carbon ions used in hadrontherapy. In particular, although almost identical relative depth dose profiles were observed for carbon and neon ion beams, the $\overline{{L_{d} }}$ and the $\overline{{y_{d} }}$ depth-profiles displayed that values 2 to 3 times higher were found for neon ion beams. Furthermore, neon ion *yd(y)* spectra exhibited the narrowest shape amongst all the beams evaluated, with lineal energies extending over a single order of magnitude up to values as high as 2000 keV/µm. This suggests high probability of lethal events and increased biological efficacy in comparison to carbon ions. A slightly lower relative efficacy of neon over carbon beams have been noticed in the Bragg peak region as compared to the plateau region, potentially due to an increased overkilling effect for the broad beam neon-ions. At a given LET and particle type, our RBE_10_ calculations were found to be compatible with experimental RBE values found in literature, some performed with the same HSG cell lines^53,54^. Although we have only displayed the RBE values calculated at 4 and 8.2 cm in depth in Table 1, we were able to verify that the RBE values were, as expected, increasing with the LET of particles, as the range of LETs covered in this study varied from 5 to 800 keV/µm according to the depth and the type of ion. It is well established that higher LET will be associated with more complex damage, such as clustered DNA breaks which are much more difficult to repair and result in a higher RBE (except when other effects comes into play such as the overkill effect as discussed above). Particularly for protons, considering a constant RBE of 1.1 along its entire range would lead to potential errors in the evaluation of the biological dose delivered in a treatment, as it has been well described in the literature^52^. Finally, maximum $\overline{{L_{d} }}$ and $\overline{{y_{d} }}$ values were found at the same position as the dose maximum for neon ions, while these maximums were shifted to a higher depth for the proton ion in particular, which could be an advantage for future treatment planning optimizations as good coherence is to be expected between classically used dose and biologically-relevant quantities.

This MC study represents a first step towards a full evaluation of the biological efficacy of VHEE beams. It brings additional arguments forward in favor of performing cellular and in vivo biological experiments to explore the effective radiobiological response of living tissues to VHEEs. Both approaches concluded that no, or little, potential higher biological efficacy is to be expected from VHEE beams. The increased probability of nuclear reactions due to the increased electron energy did not lead to significant consequences in terms of the biologically-relevant calculated quantities. This is a very important result moving forward towards the clinical use of such beams as they should not incur additional detrimental effects over clinically used electron beams, thus allowing their dosimetry to be planned similarly. In addition, VHEEs are of particular interest for FLASH radiotherapy, as they should allow the treatment of deep-seated tumors while hopefully providing better protection to the healthy tissues in the beam path compared to using conventional dose-rates. With this in mind, already having access to the theoretical data is then of great interest, as it is highly probable that the first sets of in vivo experimental data being acquired with VHEEs would be under the FLASH regime, thus bringing additional biological mechanisms to the forefront over a conventional change of particle type.
